# Supplementary material for: Sex differences, asymmetry, and age‐related white matter development in infants and 5‐year‐olds as assessed with tract‐based spatial statistics
Source: Hum Brain Mapp. 2023 Mar 22;44(7):2712–25. doi: 10.1002/hbm.26238 (PMC10089102; doi:10.1002/hbm.26238)
Supplement: Supplementary file 1 — Data S1. Supporting Information [file HBM-44-2712-s001.pdf]

Supplementary material to

Sex differences, asymmetry and age-related white matter development in infants and 5-year-olds as assessed with Tract-Based Spatial Statistics

Authors: Venla Kumpulainen[a], Harri Merisaari[a,b], Eero Silver[a] #, Anni Copeland[a] #, Elmo P. Pulli[a] #, John D Lewis[c], Ekaterina Saukko[b], Satu J. Shulist[a], Jani Saunavaara[d], Riitta Parkkola[a,b], Tuire Lähdesmäki[a,e], Linnea Karlsson[a,f,g,h] , Hasse Karlsson[a,g,h] , Jetro J. Tuulari[a, g,h,i,j]

# shared contribution

a FinnBrain Birth Cohort Study, Turku Brain and Mind Center, Department of Clinical Medicine, University of Turku, Turku, Finland

b Department of Radiology, Turku University Hospital, Turku, Finland

c Montreal Neurological Institute, McGill University, Montreal, Canada

d Department of Medical Physics, Turku University Hospital and University of Turku, Turku Finland

e Department of Pediatric Neurology, Turku University Hospital, University of Turku, Turku, Finland

f Department of Paediatrics and Adolescent Medicine, Turku University Hospital and University of Turku, Turku, Finland

g Department of Psychiatry, Turku University Hospital & University of Turku, Turku, Finland

h Centre for Population Health Research, Turku University Hospital and University of Turku, Turku, Finland

i Turku Collegium for Science, Medicine and Technology, University of Turku, Turku, Finland

j Department of Psychiatry, University of Oxford, Oxford, United Kingdom (Sigrid Juselius Fellowship)

Corresponding author
Venla Kumpulainen
Email veviku@utu.fi
Kiinamyllynkatu 10, FinnBrain study,
Medisiina A Building
20520 Turku
ORCID: 0000-0003-3375-5445

Supplement Table 1. Demographics of the study population. SD = standard deviation, gwk = gestational week, BMI = body-mass index, SSRI/SNRI = selective serotonine/serotonine-noradrenaline re-uptake inhibitor, EPDS = Edinburgh postnatal depressive scale, SCL = symptom checklist, mm = millimetre

**Demographics**	Infants (N = 166)	5-year-olds (N = 144)
Females (%)	77 (46)	68 (47)
Males (%)	89 (54)	76 (53)
Age (years) (SD, range)		5.38 (0.11; 5.09-5.79)
Gestational age < 35 gwk (%)	0	1 (0.69)
Gestational age at birth, days (SD, range)	279 (8.3; 265-296)	
Gestational age at scan, days (SD, range)	305 (7.2; 291-325)	
Handedness (%)		
Right		127 (88)
Left		12 (8.3)
Both		3 (2.1)
NA		2 (1.4)
At birth (SD, range)		
Weight (g)	3530 (448; 2530-4700)	3530 (520; 1790-4980)
Height (cm)	50.4 (1.88; 44-56)	50.4 (2.48; 43-56)
At the imaging (SD, range)		
Weight (kg)		21.1 (3.02; 14.0-31.0)
Height (cm)		114 (4.59; 100-125)
Ponderal index (kg/m^3)		14.0 (1.28; 11.0-18.0)
Maternal (SD, range)		
Pre-pregnancy BMI (kg/m^2)	24.4 (4.18; 17.5-40.8)	24.2 (4.10; 17.5-36.7)
NA (%)	3 (1.8)	1 (0.69)
Age at birth	30.0 (4.3; 19-41)	30.4 (4.8; 18-41)
Exposure during pregnancy (%)		
Tobacco smoking (yes/no/NA)	9(5.4)/153(92)/4(2.4)	8(5.5)/134(93)/2(1.4)
SSRI/SNRI	11(6.6)/147(89)/8(4.8)	6(4.2)/128(89)/10(6.9)
Gluco-corticosteroids	8(4.8)/144(87)/14(8.4)	14(9.7)/130(90)/0
Depressive/anxiety symptoms (SD, range)		
EPDS, 2nd trimester	5.19 (5.05; 0-25.0)	4.80 (4.22; 0-21.0)
EPDS, 3 months postpartum		4.26 (3.90; 0-19.0)
SCL-90 sum, 2nd trimester	4.12 (5.11; 0-28.0)	3.62 (3.75; 0-19.0)
SCL-90 sum, 3 months postpartum		2.55 (3.50; 0-17.0)
Framewise head displacement		
Mean rotational (°; SD, range)	0.30 (0.22; 0.06-1.64)	0.18 (0.08; 0.06-0.56)
Mean translational (mm; SD, range)	1.88 (0.48-6.61; 0.87)	1.16 (0.27; 0.64-2.26)

Supplement Table 2. Description of regression and sensitivity analyses in the current study. BMI = body-mass index, SES = socio-economic status, SSRI/SNRI = selective serotonine/serotonine-noradrenaline re-uptake inhibitor, EPDS = Edinburgh postnatal depressive scale, SCL = symptom checklist

Regression analyses	Main variable	Covariates used in sensitivity analyses
5-year-olds	• sex<br>• age<br>• handedness<br>• Ponderal index<br>• Maternal pre-pregnancy BMI<br>• Maternal sosio-economis status (by educational status, categorised high/medium to low)<br>• Smoking during pregnancy<br>• Intrascanner head motion	• Gestational age<br>• Maternal age at birth<br>• Maternal pre-pregnancy BMI<br>• Maternal SES<br>• Smoking during pregnancy<br>• Exposure to glucocorticosteroids during pregnancy<br>• Exposure to SSRI/SNRI during pregnancy<br>• EPDS at 2nd trimester<br>• EPDS 3 months postpartum<br>• SCL-90 at 2nd trimester<br>• SCL-90 3 months postpartum
Infants	• Age from birth<br>• Gestational age<br>• Sex<br>• Birth weight<br>• Maternal pre-pregnancy BMI<br>• Maternal socio-economic status<br>• Smoking during pregnancy<br>• Intrascanner head motion	• Gestational age<br>• Age from birth<br>• Maternal age at birth<br>• Maternal pre-pregnancy BMI<br>• Maternal SES<br>• Smoking during pregnancy<br>• Exposure to glucocorticosteroids during pregnancy<br>• Exposure to SSRI/SNRI during pregnancy<br>• EPDS at 2nd trimester<br>• SCL-90 at 2nd trimester

Example of sensitivity analyses

Basic model	FA = age + sex + maternal BMI
Sensitivity model 1	FA = age + sex + maternal BMI + maternal age
Sensitivity model 2	FA = age + sex + maternal BMI + SES
Sensitivity model 3	FA = age + sex + maternal BMI + smoking
Sensitivity model …	FA = age + sex + maternal BMI + …

Supplement Table 3. Mean fractional anisotropy (FA) and standard deviation (SD) of each white matter tract and differences between girls and boys. CST = corticospinal tract, ML = medial lemniscus, ICP = inferior cerebellar peduncle, SCP = superior cerebellar peduncle, CP = cerebral peduncle, ALIC = anterior limb of internal capsule, PLIC = posterior limb of internal capsule, ACR = anterior corona radiata, SCR = superior corona radiata, PCR = posterior corona radiata, PTR (OR) = posterior thalamic radiation (optic tract), ILF/IFOF = inferior longitudinal fasciculus/inferior fronto-occipital fasciculus, EC = external capsule, CG = cingulate, CING = cingulum, ST = stria terminalis, SLF = superior longitudinal fasciculus, SFOF = superior fronto-occipital fasciculus, UNC = uncinate. Left-right difference of mean FA provided, negative values denote for rightward lateralization. Statistical significance calculated with independent sample t test (two-tailed), Bonferroni corrected p value = 0.001 (0.05/36), tracts with significant difference in bold.

		Mean FA	SD	Sig.	Cohen's d
SCC	girl	0,748	0,025	0,005	0,482
	boy	0,735	0,029		
GCC	girl	0,719	0,033	0,694	0,066
	boy	0,721	0,036		
BCC	girl	0,633	0,038	0,470	0,121
	boy	0,637	0,037		
fornix	girl	0,450	0,041	0,253	0,192
	boy	0,442	0,043		
Right CST	girl	0,488	0,024	0,666	0,072
	boy	0,487	0,028		
Left CST	girl	0,504	0,027	0,288	0,178
	boy	0,509	0,028		
Right ALIC	girl	0,544	0,023	0,725	0,059
	boy	0,543	0,025		
Left ALIC	girl	0,523	0,023	0,519	0,108
	boy	0,520	0,027		
Right PLIC	girl	0,654	0,020	0,570	0,095
	boy	0,652	0,020		
Left PLIC	girl	0,657	0,021	0,952	0,010
	boy	0,657	0,021		
**Right rlIC**	**girl**	**0,553**	**0,028**	**0,001**	**0,582**
	boy	0,538	0,026		
Left rlIC	girl	0,569	0,024	0,007	0,458
	boy	0,559	0,023		
Right ACR	girl	0,458	0,028	0,150	0,242
	boy	0,452	0,028		
Left ACR	girl	0,452	0,028	0,049	0,331
	boy	0,443	0,027		
Right SCR	girl	0,471	0,024	0,120	0,261
	boy	0,464	0,025		
Left SCR	girl	0,478	0,024	0,854	0,031
	boy	0,477	0,022		
Right PCR	girl	0,444	0,025	0,056	0,321
	boy	0,436	0,028		
Left PCR	girl	0,441	0,030	0,414	0,137
	boy	0,437	0,027		

		Mean FA	SD	Sig.	Cohen's d
**Right**	**girl**	**0,587**	**0,029**	**0,000**	**0,680**
**PTR/OR**	**boy**	**0,566**	**0,032**		
**Left**	**girl**	**0,587**	**0,030**	**0,000**	**0,658**
**PTR/OR**	**boy**	**0,566**	**0,034**		
**Right**	**girl**	**0,516**	**0,028**	**0,000**	**0,644**
**ILF/IFOF**	**boy**	**0,499**	**0,026**		
**Left**	**girl**	**0,517**	**0,026**	**0,001**	**0,579**
**ILF/IFOF**	**boy**	**0,502**	**0,026**		
Right EC	girl	0,381	0,020	0,311	0,170
	boy	0,377	0,023		
Left EC	girl	0,400	0,025	0,773	0,048
	boy	0,401	0,020		
Right	girl	0,458	0,035	0,463	0,123
CING	boy	0,462	0,038		
Left	girl	0,483	0,035	0,453	0,126
CING	boy	0,488	0,039		
Right CG	girl	0,409	0,039	0,860	0,029
	boy	0,408	0,037		
Left CG	girl	0,406	0,036	0,269	0,185
	boy	0,412	0,037		
Right	girl	0,506	0,026	0,851	0,031
Fornix/ST	boy	0,505	0,032		
Left	girl	0,521	0,025	0,955	0,009
Fornix/ST	boy	0,521	0,032		
Right	girl	0,476	0,029	0,014	0,416
SLF	boy	0,465	0,025		
Left	girl	0,477	0,029	0,097	0,279
SLF	boy	0,469	0,025		
Right	girl	0,478	0,036	0,469	0,121
SFOF	boy	0,473	0,041		
Left	girl	0,446	0,037	0,709	0,062
SFOF	boy	0,449	0,045		
Right	girl	0,445	0,030	0,031	0,364
UNC	boy	0,434	0,028		
Left UNC	girl	0,453	0,035	0,194	0,218
	boy	0,447	0,028		

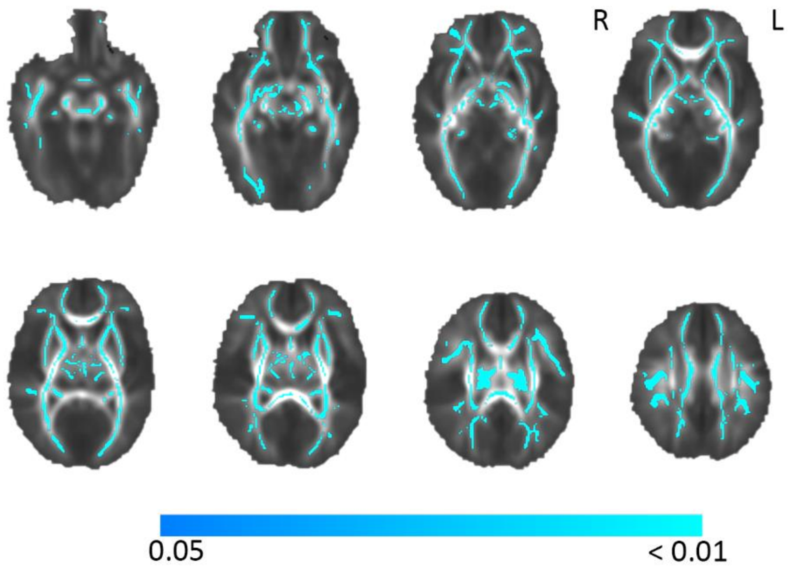

Supplement Figure 1. Effect of gestational age on fractional anisotropy analysed voxel-wise with tract-based spatial statistics with covariates (age after birth, sex, birth weight, intrascanner head motion, maternal tobacco smoking during pregnancy, socioeconomic status (low-medium/high), maternal pre-pregnancy body-mass index. Analysis conducted with 5000 permutations, threshold-free cluster enhancement correction applied, $p < 0.05$, R = right, L = left.

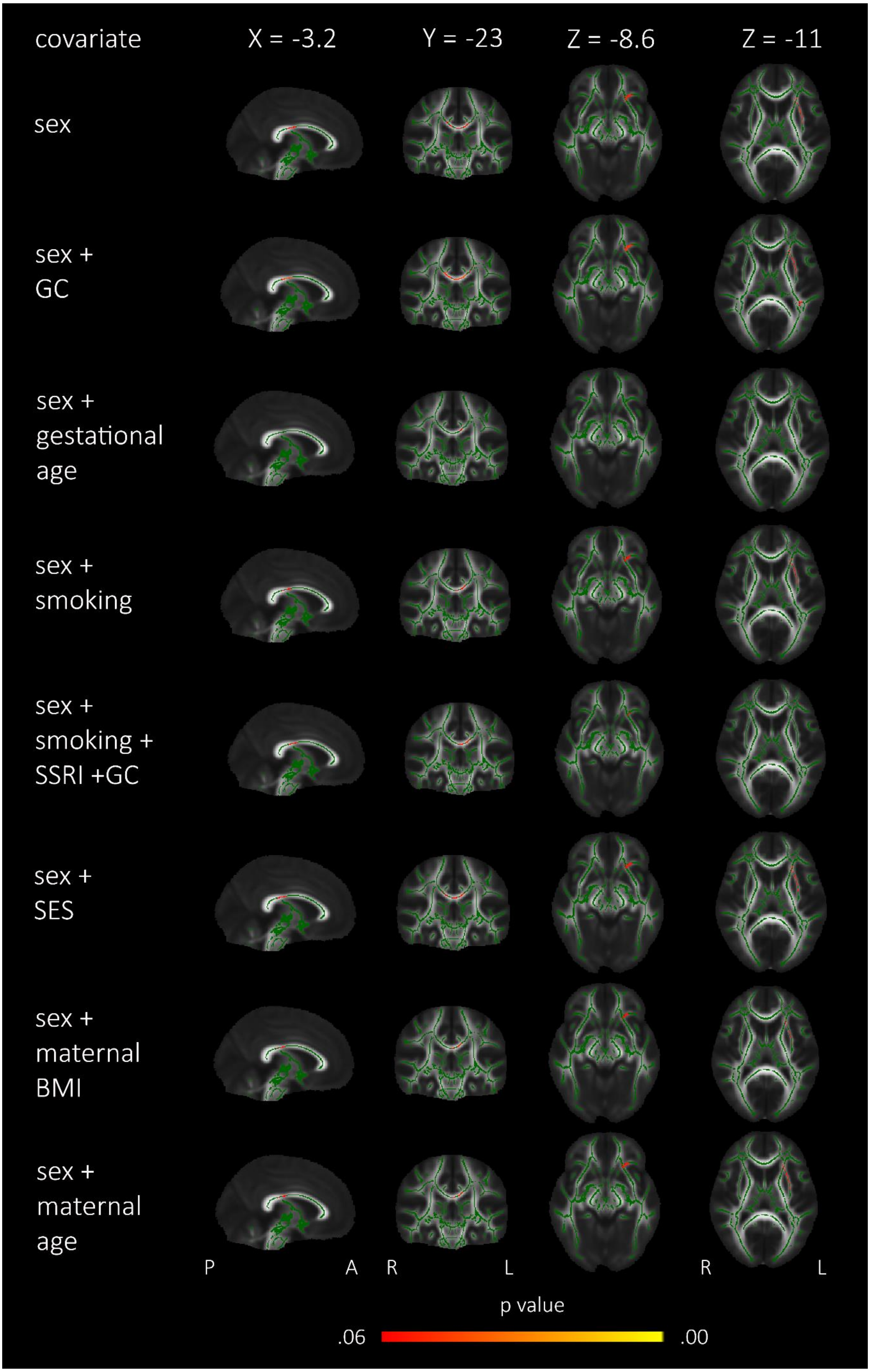
covariate
X = -3.2
Y = -23
Z = -8.6
Z = -11
sex
sex + GC
sex + gestational age
sex + smoking
sex + smoking + SSRI +GC
sex + SES
sex + maternal BMI
sex + maternal age
P
A
R
L
R
L
p value
.06
.00

Supplement Figure 2. Age-effects analysed voxel-wise with tract-based spatial statistics with covariates (prenatal glucocorticoid (GC) exposure, gestational age, maternal tobacco smoking during pregnancy, exposure to selective serotonin reuptake inhibitors (SSRI) during pregnancy, socioeconomic status (SES; low-medium/high), maternal pre-pregnancy bodymass index (BMI) and maternal age at birth). Analyses conducted with 5000 permutations, TFCE (threshold-free cluster enhancement) correction applied, $p < 0.06$.

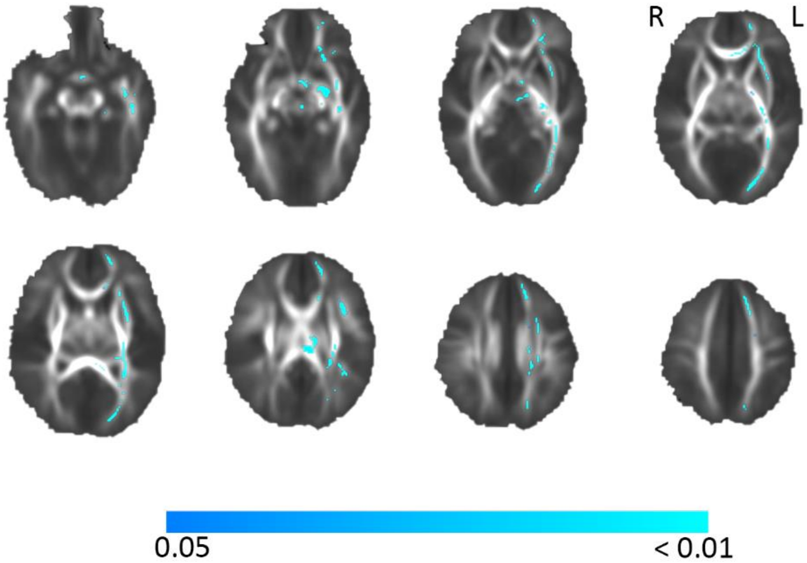

Supplement Figure 3. Lateralization pattern of white matter tract fractional anisotropy with tract-based spatial statistics in infants. Significant regions with leftward asymmetry in blue. Threshold-free cluster enhancement (TFCE) correction applied, 5000 permutations, $p < 0.05$ (blue colour bar showing p value). R = right, L = left

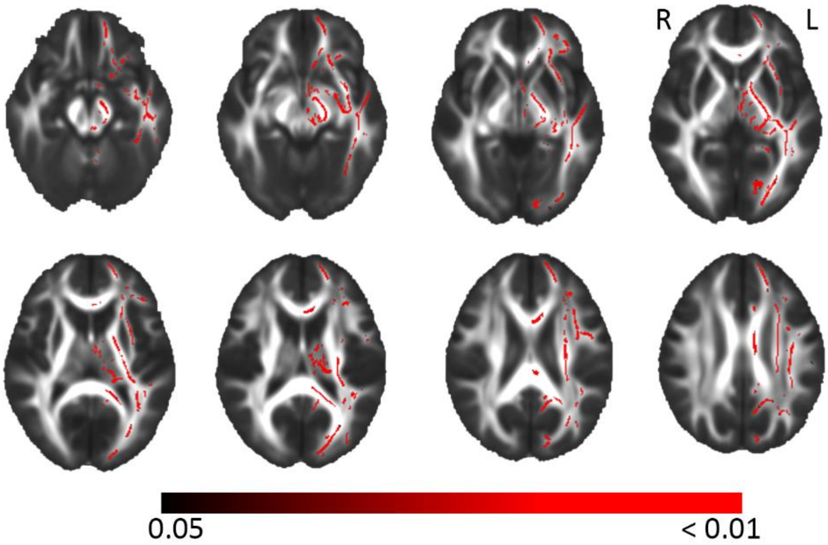

Supplement Figure 4. Lateralization pattern of white matter tract fractional anisotropy with tract-based spatial statistics in 5-year-olds. Significant regions with leftward asymmetry in red. Threshold-free cluster enhancement (TFCE) correction applied, 5000 permutations, $p < 0.05$ (red colour bar showing p value). R = right, L = left

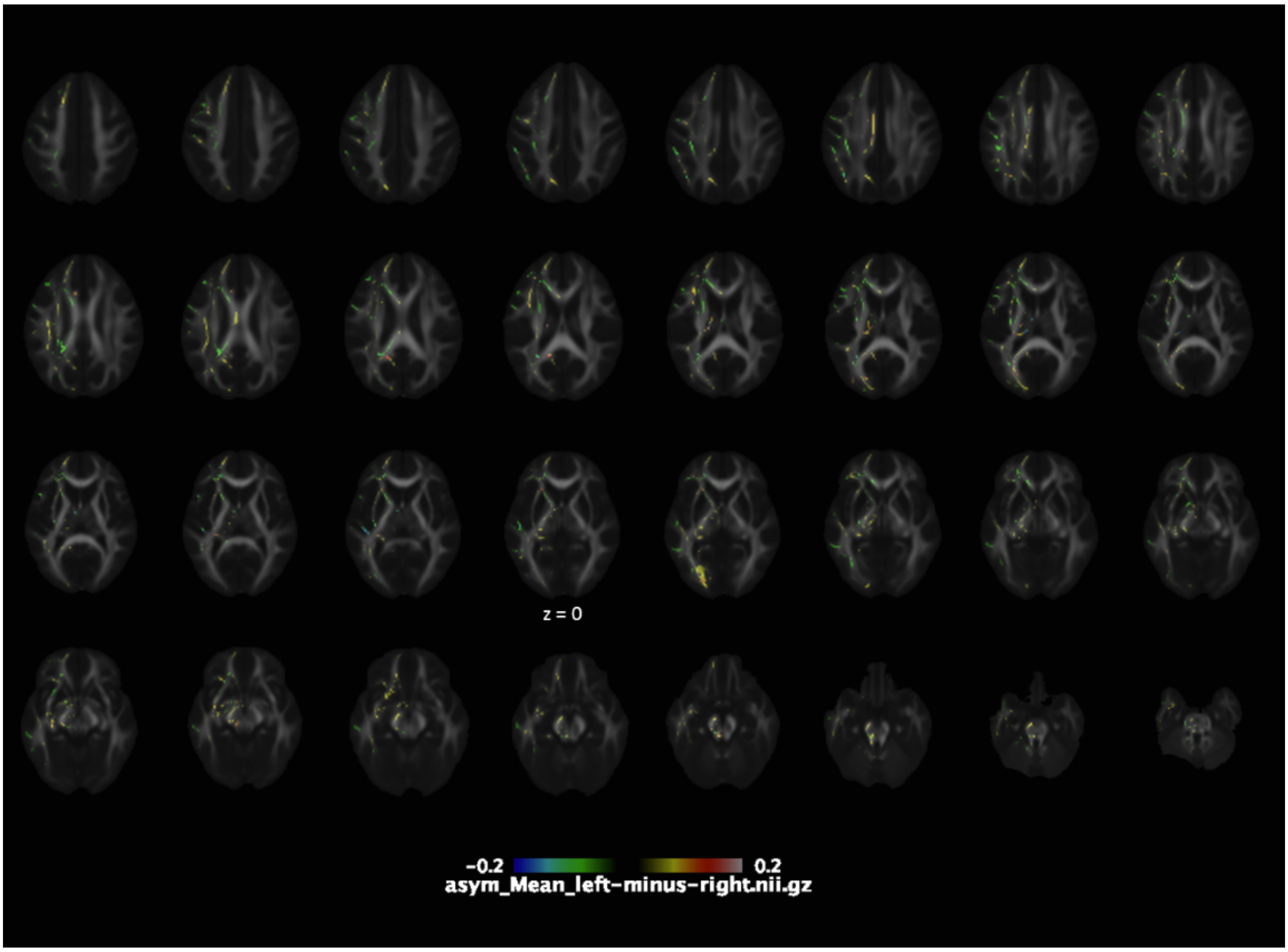

Supplement Figure 5. Mean values of lateralized FA (left minus right). Yellow to red colours (positive values) indicate leftward asymmetry and blue to green colours (negative values) rightward asymmetry.

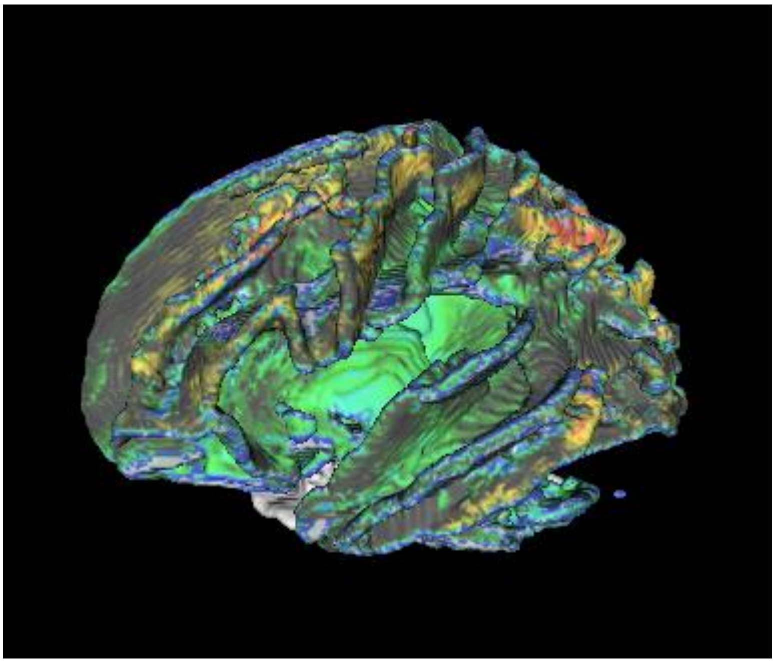

Supplement Figure 6. Yellow to red colours (positive values) indicate leftward asymmetry and blue to green colours (negative values) rightward asymmetry.

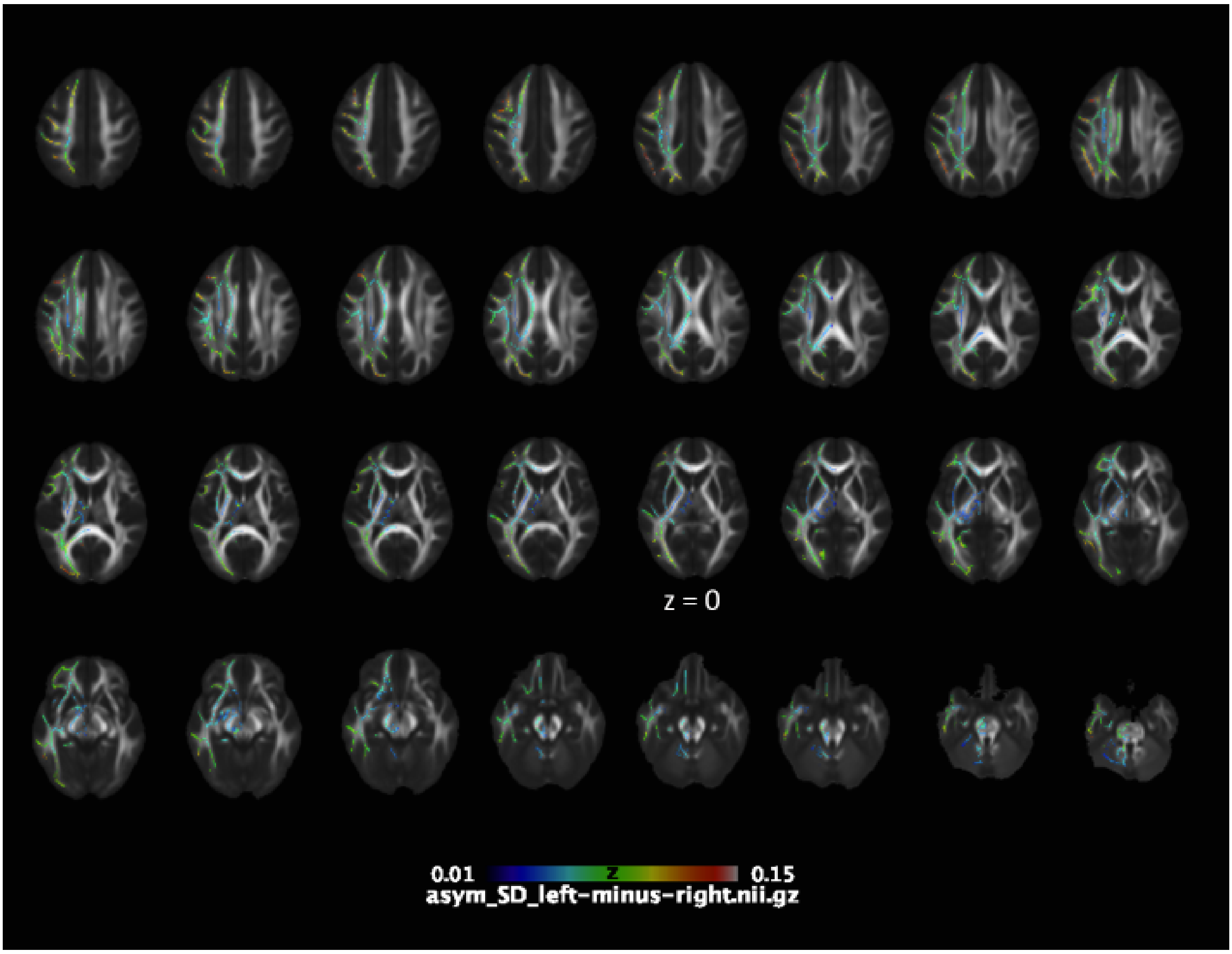

Supplement Figure 7. Inter-subject variation of lateralized FA values described as standard deviation (SD).

Supplement Table 4. Comparison of mean fractional anisotropy (FA) values in bilateral white matter tracts in 5-year-olds. CST = corticospinal tract, ML = medial lemniscus, ICP = inferior cerebellar peduncle, SCP = superior cerebellar peduncle, CP = cerebral peduncle, ALIC = anterior limb of internal capsule, PLIC = posterior limb of internal capsule, ACR = anterior corona radiata, SCR = superior corona radiata, PCR = posterior corona radiata, PTR (OR) = posterior thalamic radiation (optic tract), ILF/IFOF = inferior longitudinal fasciculus/inferior fronto-occipital fasciculus, EC = external capsule, CG = cingulate, CING = cingulum, ST = stria terminalis, SLF = superior longitudinal fasciculus, SFOF = superior fronto-occipital fasciculus, UNC = uncinate. Left-right difference of mean FA provided, negative values denote for rightward lateralization. Statistical significance calculated with one sample t test (two-tailed), Bonferroni corrected p value = 0.001 (0.05/36), tracts with significant difference in bold.

Tract		Mean FA	SD	Left-right difference	Sig.	Cohen's d
**CST**	**right**	**0,487**	**0,026**	**0,0194**	**0,000**	**0,721**
	**left**	**0,507**	**0,028**			
**ALIC**	**right**	**0,544**	**0,024**	**-0,0224**	**0,000**	**0,903**
	**left**	**0,521**	**0,025**			
PLIC	right	0,653	0,020	0,0041	0,087	0,202
	left	0,657	0,021			
ACR	right	0,455	0,028	-0,0077	0,019	0,278
	left	0,447	0,028			
**SCR**	**right**	**0,467**	**0,025**	**0,0100**	**0,000**	**0,420**
	left	0,477	0,023			
PCR	right	0,440	0,027	-0,0006	0,843	0,023
	left	0,439	0,028			
PTR/OR	right	0,576	0,032	-0,0002	0,951	0,007
	left	0,576	0,034			
ILF/IFOF	right	0,507	0,028	0,0021	0,515	0,077
	left	0,509	0,027			
**EC**	**right**	**0,379**	**0,022**	**0,0220**	**0,000**	**1,00**
	left	0,401	0,022			
**CING**	**right**	**0,460**	**0,036**	**0,0256**	**0,000**	**0,700**
	left	0,485	0,037			
CG	right	0,408	0,038	0,0010	0,822	0,026
	left	0,409	0,036			
SLF	right	0,470	0,027	0,0027	0,403	0,099
	left	0,473	0,027			
**SFOF**	**right**	**0,475**	**0,039**	**-0,0281**	**0,000**	**0,706**
	**left**	**0,447**	**0,041**			
UNC	right	0,439	0,029	0,0106	0,003	0,350
	left	0,450	0,031			

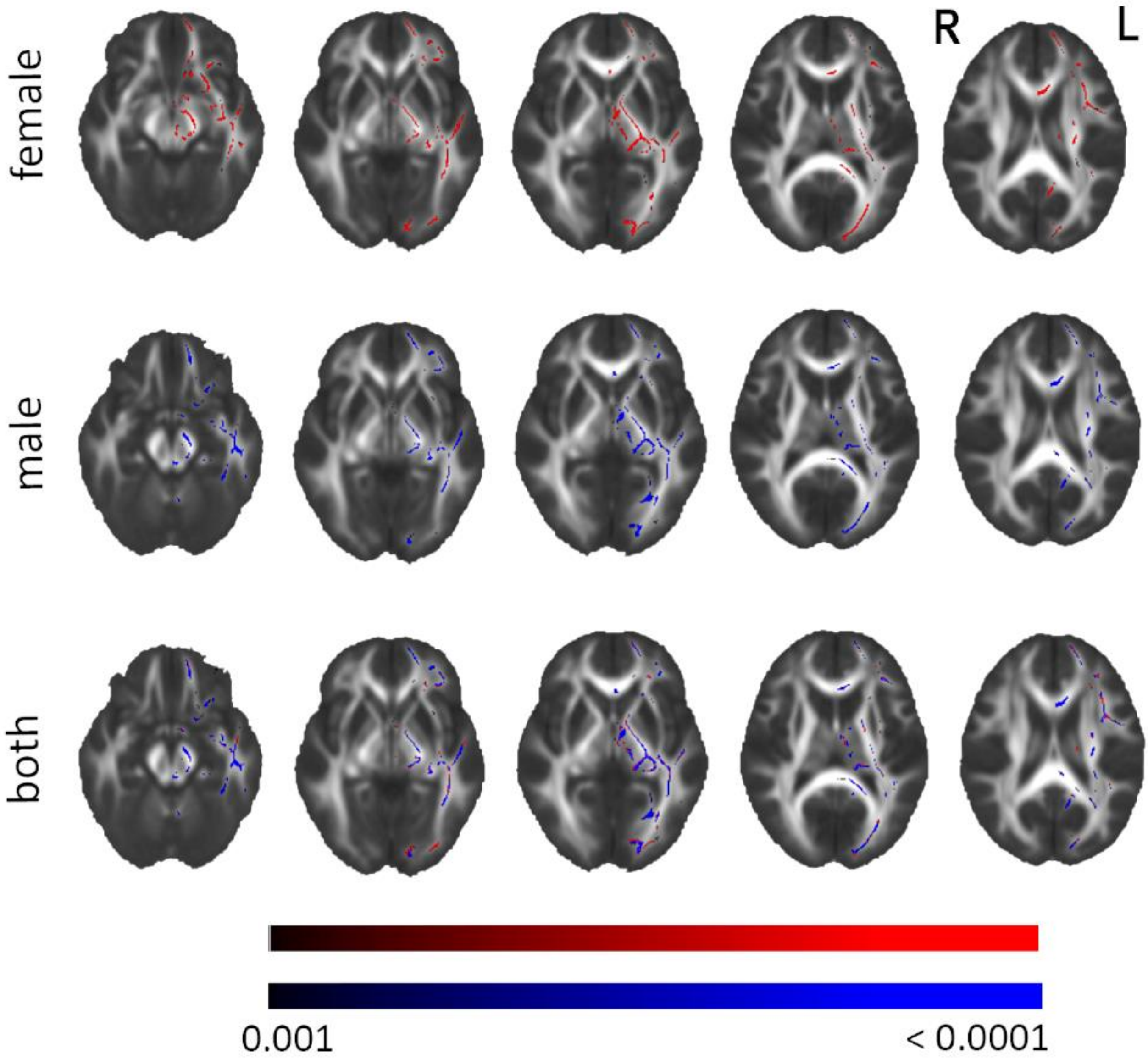

Supplement Figure 8. Sex-specific white matter lateralization patterns of 5-year-olds with Tract-based Spatial Statistical (TBSS) analysis, depicted as left-right fractional anisotropy values. The statistically significantly asymmetric regions showed with red in females and with blue in males. Analyses conducted with 5000 permutations, threshold-free cluster enhancement (TFCE) correction applied, p < 0.001. R = right, L = left

Supplement Table 5. Mean fractional anisotropy (FA) and standard deviation (SD) of each white matter tract in infants and in 5-year-olds. GCC = genu of corpus callosum, BCC = body of corpus callosum, SCC = splenium of corpus callosum, CST = corticospinal tract, ICP = inferior cerebellar peduncle, SCP = superior cerebellar peduncle, CP = cerebral peduncle, ALIC = anterior limb of internal capsule, PLIC = posterior limb of internal capsule, rl IC = retrolenticular internal capsule, ACR = anterior corona radiata, SCR = superior corona radiata, PCR = posterior corona radiata, PTR (OR) = posterior thalamic radiation (optic tract), ILF/IFOF = inferior longitudinal fasciculus/inferior fronto-occipital fasciculus, EC = external capsule, CG = cingulate, CING = cingulum, ST = stria terminalis, SLF = superior longitudinal fasciculus, SFOF = superior fronto-occipital fasciculus, UNC = uncinate, R = right, L = left.

	infants		5-year-olds	
	FA	SD	FA	SD
GCC	0.33	0.029	0.72	0.035
BCC	0.31	0.026	0.64	0.038
SCC	0.39	0.032	0.74	0.028
Fornix	0.24	0.020	0.45	0.042
CST R	0.24	0.024	0.49	0.026
CST L	0.24	0.027	0.51	0.028
ICP R	0.20	0.020	0.47	0.028
ICP L	0.21	0.026	0.47	0.024
SCP R	0.27	0.024	0.57	0.028
SCP L	0.32	0.028	0.60	0.028
CP R	0.31	0.023	0.63	0.024
CP L	0.32	0.027	0.64	0.024
ALIC R	0.29	0.020	0.54	0.024
ALIC L	0.26	0.020	0.52	0.025
PLIC R	0.39	0.024	0.65	0.020
PLIC R	0.38	0.024	0.66	0.021
rl IC R	0.38	0.024	0.54	0.028
rl IC L	0.36	0.022	0.56	0.024
ACR R	0.22	0.026	0.46	0.028
ACR L	0.22	0.025	0.45	0.028
SCR R	0.29	0.022	0.47	0.025
SCR L	0.28	0.021	0.48	0.023
PCR R	0.25	0.025	0.44	0.027
PCR L	0.27	0.026	0.44	0.028
PTR/OR R	0.31	0.024	0.58	0.032
PTR/OR L	0.32	0.026	0.58	0.034
ILF/IFOF R	0.29	0.027	0.51	0.028
ILF/IFOF L	0.30	0.027	0.51	0.027
EC R	0.24	0.017	0.38	0.022
EC L	0.25	0.017	0.40	0.022
CG R	0.25	0.043	0.46	0.036
CG L	0.27	0.035	0.49	0.037
CING R	0.19	0.026	0.41	0.038
CING L	0.20	0.026	0.41	0.036
Fornix/ST R	0.26	0.021	0.51	0.029
Fornix/ST L	0.31	0.023	0.52	0.028
SLF R	0.23	0.023	0.47	0.027
SLF L	0.24	0.022	0.47	0.027
SFOF R	0.34	0.030	0.48	0.039
SFOF L	0.19	0.020	0.45	0.041

UNC R	0.30	0.045	0.44	0.029
UNC L	0.26	0.051	0.45	0.031

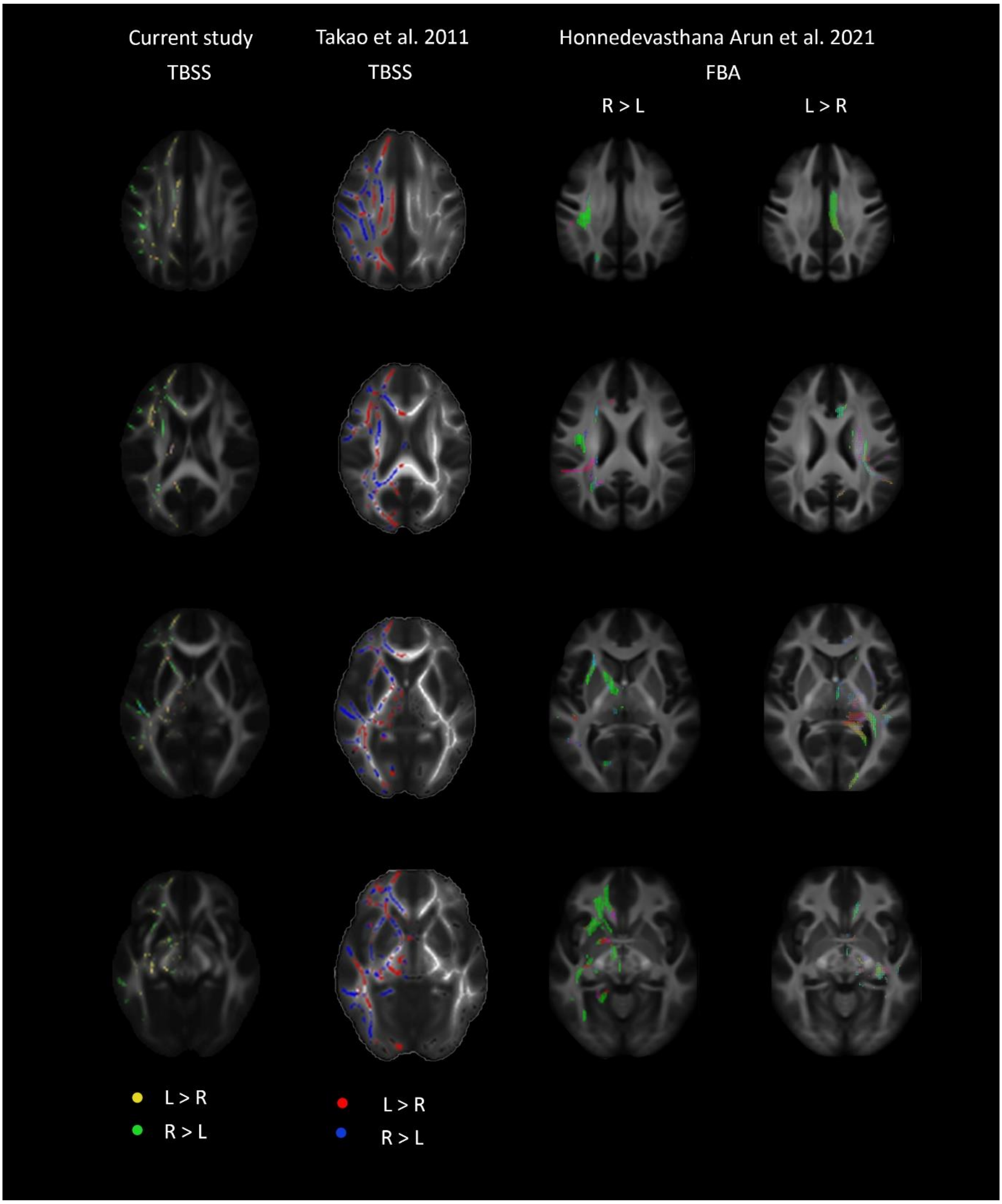

Supplement Figure 9. Comparison of lateralization patterns in our study and two previously published adult-population studies (Takao et al. 2011 and Honnedevasthana Arun et al. 2021). Figure adapted from previously published figures by Takao et al. 2011[1] and Honnedevasthana Arun et al. 2021[2], Copyright Elsevier. TBSS = tract-based spatial statistics, FBA = fixel-based analysis, L = left, R = right

1. Takao, H., Hayashi, N., & Ohtomo, K. (2011). White matter asymmetry in healthy individuals: A diffusion tensor imaging study using tract-based spatial statistics. *Neuroscience*, *193*, 291–299. https://doi.org/10.1016/j.neuroscience.2011.07.041

2. Honnedevasthana Arun, A., Connelly, A., Smith, R. E., & Calamante, F. (2021). Characterisation of white matter asymmetries in the healthy human brain using diffusion MRI fixel-based analysis. *NeuroImage*, *225*(November 2020), 117505. https://doi.org/10.1016/j.neuroimage.2020.117505
